# When are hypotheses useful in ecology and evolution?

**DOI:** 10.1002/ece3.7365

**Published:** 2021-03-25

**Authors:** Matthew G. Betts, Adam S. Hadley, David W. Frey, Sarah J. K. Frey, Dusty Gannon, Scott H. Harris, Hankyu Kim, Urs G. Kormann, Kara Leimberger, Katie Moriarty, Joseph M. Northrup, Ben Phalan, Josée S. Rousseau, Thomas D. Stokely, Jonathon J. Valente, Chris Wolf, Diego Zárrate‐Charry

**Affiliations:** ^1^ Forest Biodiversity Research Network Department of Forest Ecosystems and Society Oregon State University Corvallis OR USA; ^2^ USDA Forest Service Pacific Northwest Research Station Corvallis OR USA; ^3^ Wildlife Research and Monitoring Section Ontario Ministry of Natural Resources and Forestry Environmental and Life Sciences Graduate Program Trent University Peterborough ON Canada

**Keywords:** hypothesis, mechanisms, multiple working hypotheses, prediction, scientific method

## Abstract

Research hypotheses have been a cornerstone of science since before Galileo. Many have argued that hypotheses (1) encourage discovery of mechanisms, and (2) reduce bias—both features that should increase transferability and reproducibility. However, we are entering a new era of big data and highly predictive models where some argue the hypothesis is outmoded. We hypothesized that hypothesis use has declined in ecology and evolution since the 1990s, given the substantial advancement of tools further facilitating descriptive, correlative research. Alternatively, hypothesis use may have become *more* frequent due to the strong recommendation by some journals and funding agencies that submissions have hypothesis statements. Using a detailed literature analysis (*N* = 268 articles), we found prevalence of hypotheses in eco–evo research is very low (6.7%–26%) and static from 1990–2015, a pattern mirrored in an extensive literature search (*N* = 302,558 articles). Our literature review also indicates that neither grant success nor citation rates were related to the inclusion of hypotheses, which may provide disincentive for hypothesis formulation. Here, we review common justifications for avoiding hypotheses and present new arguments based on benefits to the individual researcher. We argue that stating multiple alternative hypotheses increases research clarity and precision, and is more likely to address the mechanisms for observed patterns in nature. Although hypotheses are not always necessary, we expect their continued and increased use will help our fields move toward greater understanding, reproducibility, prediction, and effective conservation of nature.

## Graphical abstract

We use a quantitative literature review to show that use of a priori hypotheses is still rare in the fields of ecology and evolution. We provide suggestions about the group and individual‐level benefits of hypothesis use.

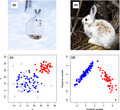

## 1 INTRODUCTION

Why should ecologists have hypotheses? At the beginning of most science careers, there comes a time of “hypothesis angst” where students question the need for the hypothetico‐deductive approach their elders have deemed essential for good science. Why is it not sufficient to just have a research objective or question? Why can't we just collect observations and describe those in our research papers?

Research hypotheses are explanations for an observed phenomenon (Loehle, 1987; Wolff & Krebs, 2008) (see Box 1) and have been proposed as a central tool of science since Galileo and Francis Bacon in the mid‐1600s (Glass & Hall, 2008). Over the past century, there have been repeated calls for rigorous application of hypotheses in science, and arguments that hypothesis use is the cornerstone of the scientific method (Chamberlin, 1890; Popper, 1959; Romesburg, 1981). In a seminal paper in *Science,* Platt (1964) challenged all scientific fields to adopt and rigorously test multiple hypotheses (sensu Chamberlin, 1890), arguing that without such hypothesis tests, disciplines would be prone to “stamp collecting” (Landy, 1986). To constitute “strong inference,” Platt required the scientific method to be a three‐step process including (1) developing alternative hypotheses, (2) devising a set of “crucial” experiments to eliminate all but one hypothesis, and (3) performing the experiments (Elliott & Brook, 2007).

BOX 1 **Definitions of hypotheses and associated terms**

**Hypothesis**: An explanation for an observed phenomenon.

**Research Hypothesis:** A statement about a phenomenon that also includes the potential mechanism or cause of that phenomenon. Though a research hypothesis doesn't need to adhere to this strict framework it is often best described as the “if” in an “if‐then” statement. In other words, “if X is true” (where X is the mechanism or cause for an observed phenomenon) “then Y” (where Y is the outcome of a crucial test that supports the hypothesis). These can also be thought of as “**mechanistic hypotheses**” since they link with a causal mechanism. For example, trees grow slowly at high elevation because of nutrient limitation (hypothesis); if this is the case, fertilizing trees should result in more rapid growth (prediction).

**Prediction:** The potential outcome of a test that would support a hypothesis. Most researchers call the second part of the if‐then statement a “prediction”.

**Multiple alternative hypotheses:** Multiple plausible explanations for the same phenomenon.

**Descriptive Hypothesis:** Descriptive statements or predictions with the word “hypothesis” in front of them. Typically researchers state their guess about the results they expect and call this the “hypothesis” (e.g., “I hypothesize trees at higher elevation will grow slowly”).

**Statistical Hypothesis**: A predicted pattern in data that should occur if a research hypothesis is true.

**Null Hypothesis**: A concise statement expressing the concept of “no difference” between a sample and the population mean.

**FIGURE 1:**
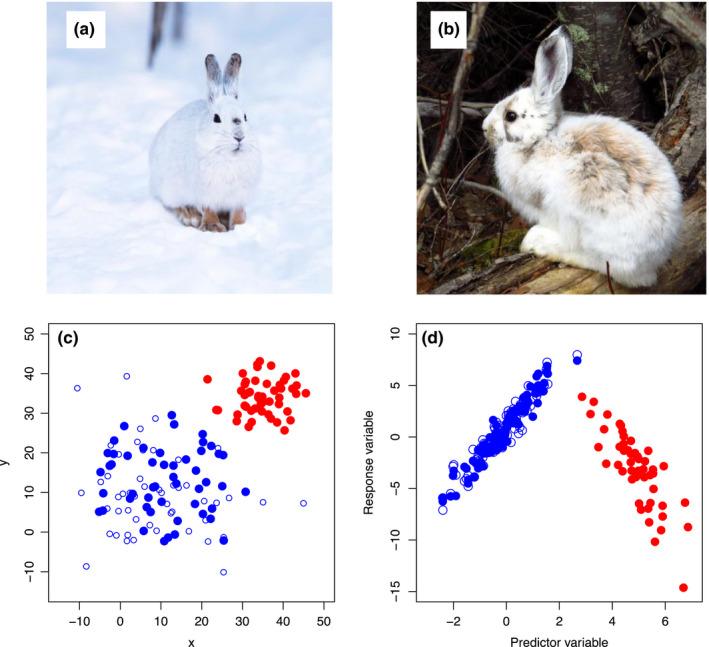
Understanding mechanisms often increases model transferability. Panels (a and b) show snowshoe hares in winter and summer coloration, respectively. If a correlative (i.e., nonmechanistic) model for hare survival as a function of color was trained only on hares during the winter and then extrapolated into the summer months, it would perform poorly (white hares would die disproportionately under no‐snow conditions). On the other hand, a researcher testing mechanisms for hare survival would (ideally via experimentation) arrive at the conclusion that it is not the whiteness of hares, but rather blending with the background that confers survival (the “camouflage” hypothesis). Understanding mechanism results in model predictions being robust to novel conditions. Panel (c) Shows *x* and *y* geographic locations of training (blue filled circles) and testing (blue open circles) locations for a hypothetical correlative model. Even if the model performs well on these independent test data (predicting open to closed circles), there is no guarantee that it will predict well outside of the spatial bounds of the existing data (red circles). Nonstationarity (in this case caused by a nonlinear relationship between predictor and response variable; panel d) could result in correlative relationships shifting substantially if extrapolated to new times or places. However, mechanistic hypotheses aimed at understanding the underlying factors driving the distribution of this species would be more likely to elucidate this nonlinear relationship. In both of these examples, understanding drivers behind ecological patterns—via testing mechanistic hypotheses—is likely to enhance model transferability

The commonly touted strengths of hypotheses are two‐fold. First, by adopting multiple plausible explanations for a phenomenon (hereafter “*multiple alternative hypotheses*”; Box 1), a researcher reduces the chance that they will become attached to a single possibility, thereby biasing research in favor of this outcome (Chamberlin, 1890); this “confirmation bias” is a well‐known human trait (Loehle, 1987; Rosen, 2016) and likely decreases reproducibility (Munafò et al., 2017). Second, various authors have argued that the a priori hypothesis framework forces one to think in advance about—and then test—various *causes* for patterns in nature (Wolff & Krebs, 2008), rather than simply examining the patterns themselves and coming up with explanations after the fact (so called “inductive research;” Romesburg, 1981). By understanding and testing mechanisms, science becomes more reliable and transferable (Ayres & Lombardero, 2017; Houlahan et al., 2017; Sutherland et al., 2013) (Figure 1). Importantly, both of these strengths should have strong, positive impacts on reproducibility of ecological and evolutionary studies (see Discussion).

However, we are entering a new era of ecological and evolutionary science that is characterized by massive datasets on genomes, species distributions, climate, land cover, and other remotely sensed information (e.g., bioacoustics, camera traps; Pettorelli et al., 2017). Exceptional computing power and new statistical and machine‐learning algorithms now enable thousands of statistical models to be run in minutes. Such datasets and methods allow for pattern recognition at unprecedented spatial scales and for huge numbers of taxa and processes. Indeed, there have been recent arguments in both the scientific literature and popular press to do away with the traditional scientific method and a priori hypotheses (Glass & Hall, 2008; Golub, 2010). These arguments go something along the lines of “if we can get predictions right most of the time, why do we need to know the cause?”

In this paper, we sought to understand if hypothesis use in ecology and evolution has shifted in response to these pressures on the discipline. We, therefore, hypothesized that hypothesis use has declined in ecology and evolution since the 1990s, given the substantial advancement of tools further facilitating descriptive, correlative research (e.g., Cutler et al., 2007; Elith et al., 2008). We predicted that this decline should be particularly evident in the applied conservation literature—where the emergence of machine‐learning models has resulted in an explosion of conservation‐oriented species distribution models (Elith et al., 2006). Our alternative hypothesis was that hypothesis use has become more frequent. The mechanism for such increases is that higher‐profile journals (e.g., *Functional Ecology*, *Proceedings of the Royal Society of London Ser. B*) and competitive granting agencies (e.g., the U.S. National Science Foundation) now require or strongly encourage hypothesis statements.

As noted above, many have argued that hypotheses are useful and important for overall progress in science, because they facilitate the discovery of mechanisms, reduce bias, and increase reproducibility (Platt, 1964). However, for hypothesis use to be propagated among scientists, one would also expect hypotheses to confer benefits to the individual. We, therefore, tested whether hypothesis use was associated with individual‐level incentives relevant to academic success: publications, citations, and grants (Weinberg, 2010). If hypothesis use confers individual‐level advantages, then hypothesis‐based research should be (1) published in more highly ranked journals, (2) have higher citation rates, and (3) be supported by highly competitive funding sources.

Finally, we also present some common justifications for absence of hypotheses and suggest potential counterpoints researchers should consider prior to dismissing hypothesis use, including potential benefits to the individual researcher. We hope this communication provides practical recommendations for improving hypothesis use in ecology and evolution—particularly for new practitioners in the field (Box 2).

BOX 2 **Recommendations for improving hypotheses use in ecology and evolution**

**Authors**: Know that you are human and prone to confirmation bias and highly effective at false pattern recognition. Thus, inductive research and single working hypotheses should be rare in your research. Remember that if your work is to have a real “impact”, it needs to withstand multiple tests from other labs over the coming decades.

**Editors and Reviewers**: Reward research that is conducted using principles of sound scientific method. Be skeptical of research that smacks of data dredging, *post hoc* hypothesis development, and single hypotheses. If no hypotheses are stated in a paper and/or the paper is purely descriptive, ask whether the novelty of the system and question warrant this, or if the field would have been better served by a study with mechanistic hypotheses. If only single hypotheses are stated, ask whether appropriate precautions were taken for the researcher to avoid finding support for a pet idea (e.g., blinded experiments, randomized attribution of treatments, etc.). To paraphrase Platt (1964): beware of the person with only one method or one instrument, either experimental or theoretical.

**Mentors**: Encourage your advisees to think carefully about hypothesis use and teach them how to construct sound multiple, mechanistic hypotheses. Importantly, explain why hypotheses are important to the scientific method, the individual and group consequences of excluding them, and the rare instances where they may not be necessary.

**Policymakers/media/educators/students/readers**: Read scientific articles with skepticism; have a scrutinous eye out for single hypothesis studies and p‐hacking. Reward multi‐hypothesis, mechanistic, predictive science by giving it greater weight in policy decisions (Sutherland et al., 2013), more coverage in the media, greater leverage in education, and more citations in reports.

## 2 METHODS

### 2.1 Literature analysis

To examine hypothesis use over time and test whether hypothesis presence was associated with research type (basic vs. applied), journal impact factor, citation rates, and grants, we sampled the ecology and evolution literature using a stratified random sample of ecology and evolution journals in existence before 1991. First, we randomly selected 19 journals across impact factor (IF) strata ranging from 0.5–10.0 in two bins (<3 IF and ≥3 IF; see Figure 3 for full journal list). We then added three multidisciplinary journals that regularly publish ecology and evolution articles (*Proceedings of the National Academy of Sciences, Science, and Nature*). From this sample of 22 journals, we randomly selected ecology and evolution articles within 5‐year strata beginning in 1991 (3 articles/journal per 5‐year bin) to ensure the full date range was evenly sampled. We removed articles in the following categories: editorials, corrections, reviews, opinions, and methods papers. In multidisciplinary journals, we examined only ecology, evolution, and conservation biology articles, as indicated by section headers in each journal. Once selected, articles were randomly distributed to the authors of the current paper (hereafter “reviewers:” MGB, ASH, DF, SF, DG, SH, HK, UK, KL, KM, JN, BP, JSR, TSS, JV, DZC) for detailed examination. On rare occasions, an article was not found, or reviewers were not able to complete their review. Ultimately, our final sample comprised 268 articles.

Reviewers were given a maximum of 10 min to find research hypothesis statements within the abstract or introduction of articles. We chose 10 min to simulate the amount of time that a journal editor pressed for time might spend evaluating the introductory material in an article. After this initial 10 min period, we determined: (1) whether or not an article contained at least one hypothesis, (2) whether hypotheses were mechanistic or not (i.e., the authors claimed to examine the mechanism for an observed phenomenon), (3) whether multiple alternative hypotheses were considered (sensu Chamberlin, 1890), and (4) whether hypotheses were “descriptive” (that is, they did not explore a mechanism but simply stated the expected direction of an effect; we define this as a “prediction” [Box 1]). It is important to note that to be identified as having hypotheses, articles did not need to contain the actual term “hypothesis” under our protocol; we also included articles using phrases such as “If X is true, *we expected*…” or “*we anticipated,*” both of which reflect a priori expectations from the data. We categorized each article as either basic (fundamental research without applications as a focus) or applied (clear management or conservation focus to article). Finally, we also examined all articles for funding sources and noted the presence of a national or international‐level competitive grant (e.g., National Science Foundation, European Union, Natural Sciences and Engineering Research Council). We assumed that published articles would have fidelity to the hypotheses stated in original grant proposals that funded the research, therefore, the acknowledgment of a successful grant is an indicator of financial reward for including hypotheses in initial proposals. Journal impact factors and individual article citation rates were gleaned directly from Web of Science. We reasoned that many researchers seek out journals with higher impact factors for the first submission of their manuscripts (Paine & Fox, 2018). Our assumption was that studies with more careful experimental design—including hypotheses—should be published where initially submitted, whereas those without may be eventually published, on average, in lower impact journals (Opthof et al., 2000). Ideally, we could have included articles that were rejected and never published in our analysis, but such articles are notoriously difficult to track (Thornton & Lee, 2000).

To support our detailed literature analysis, we also tested for temporal trends in hypothesis use within a broader sample of the ecology and evolution literature. For the same set of 22 journals in our detailed sample, we conducted a Web of Science search for articles containing “hypoth*” in the title or abstract. To calculate the proportion of articles with hypotheses (from 1990–2018), we divided the number of articles with hypotheses by the total number of articles (*N* = 302,558). Because our search method does not include the main text of articles and excludes more subtle ways of stating hypotheses (e.g., “We expected…,” “We predicted…”), we acknowledge that the proportion of papers identified is likely to be an underestimate of the true proportions. Nevertheless, we do not expect that the degree of underestimation would change over time, so temporal trends in the proportion of papers containing hypotheses should be unbiased.

### 2.2 Statistical analysis

We used generalized linear mixed models (GLMMs) to test for change in the prevalance of various hypothesis types over time (descriptive, mechanistic, multiple, any hypothesis). Presence of a hypothesis was modeled as dichotomous (0,1) with binomial error structure, and “journal” was included as a random effect to account for potential lack of independence among articles published in the same outlet. The predictor variable (i.e., year) was scaled to enable convergence. Similarly, we tested for differences in hypothesis prevalence between basic and applied articles using GLMMs with “journal” as a random effect. Finally, we tested the hypothesis that hypothesis use might decline over time due to the emergence of machine‐learning in the applied conservation literature; specifically, we modeled “hypothesis presence” as a function of the statistical interaction between “year” and “basic versus applied” articles. We conducted this test for all hypothesis types. GLMMs were implemented in R (version 3.60) using the lme4 package (Bates et al., 2018). In three of our models, the “journal” random effect standard deviation was estimated to be zero or nearly zero (i.e., 10^–8^). In such cases, the model with the random effect is exceptionally difficult to estimate, and the random effect standard deviation being estimated as approximately zero indicates the random effect was likely not needed.

We tested whether the presence of hypotheses influenced the likelihood of publication in a high‐impact journal using generalized linear models with a Gaussian error structure. We used the log of journal impact factor (+0.5) as the response variable to improve normality of model residuals. We tested the association between major competitive grants and the presence of a hypotheses using generalized linear models (logistic regression) with “hypothesis presence” (0,1) as a predictor and presence of a grant (0,1) as a response.

Finally, we tested whether hypotheses increase citation rates using linear mixed effects models (LMMs); presence of various hypotheses (0,1) were predictors in univariate models and average citations per year (log‐transformed) was the response. “Journal” was treated as a random effect, which assumes that articles within a particular journal are unlikely to be independent in their citation rates. LMMs were implemented in R using the lme4 package (Bates et al., 2015).

## 3 RESULTS

### 3.1 Trends in hypothesis use in ecology and evolution

**FIGURE 2:**
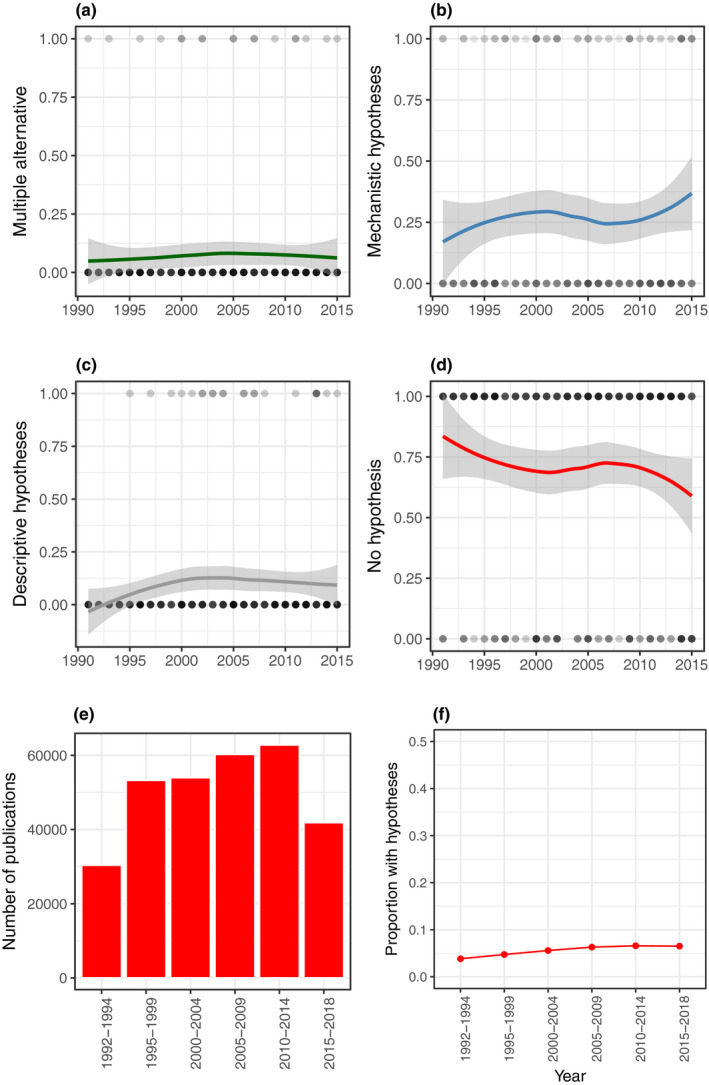
Trends in hypothesis use from 1991–2015 from a sample of the ecological and evolutionary literature (*N* = 268, (a) multiple alternative hypotheses, (b) mechanistic hypotheses, (c) descriptive hypotheses [predictions], and (d) no hypotheses present). We detected no temporal trend in any of these variables. Lines reflect LOESS smoothing with 95% confidence intervals. Dots show raw data with darker colors indicating overlapping data points. The total number of publications in ecology and evolution in selected journals has increased (e), but use of the term “hypoth*” in the title or abstracts of these 302,558 articles has remained flat, and at very low prevalence (f)

In the ecology and evolution articles we examined in detail, the prevalence of multiple alternative hypotheses (6.7%) and mechanistic hypotheses (26%) was very low and showed no temporal trend (GLMM: multiple alternative: $\hat{\beta }$ = 0.098 [95% CI: −0.383, 0.595], *z* = 0.40, *p* = 0.69, mechanistic: $\hat{\beta }$ = 0.131 [95% CI: −0.149, 0.418], *z* = 0.92, *p* = 0.36, Figure 2a,b). Descriptive hypothesis use was also low (8.5%), and although we observed a slight tendency to increase over time, 95% confidence intervals overlapped zero (GLMM: $\hat{\beta }$ = 0.351 [95% CI: −0.088, 0.819], *z* = 1.53, *p* = 0.13, Figure 2c). Although the proportion of papers containing no hypotheses appears to have declined (Figure 2d), this effect was not statistically significant (GLMM: $\hat{\beta }$ = −0.201 [95% CI: −0.483, 0.074], *z* = −1.41, *p* = 0.15). This overall pattern is consistent with a Web of Science search (*N* = 302,558 articles) for the term “hypoth*” in titles or abstracts that shows essentially no trend over the same time period (Figure 2e,f).

**FIGURE 3:**
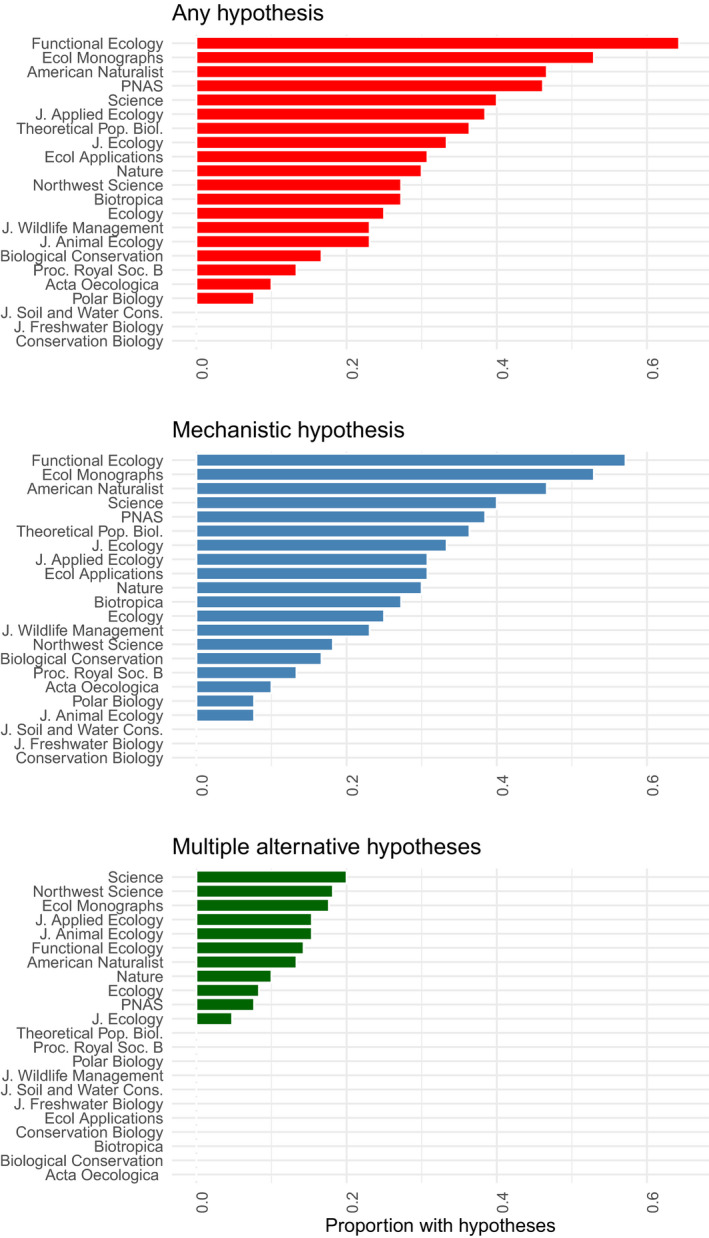
Frequency distributions showing proportion of various hypotheses types across ecology and evolution journals included in our detailed literature search. Hypothesis use varied greatly across publication outlets. We considered J. Applied Ecology, J. Wildlife Management, J. Soil, and Water Cons., Ecological Applications, Conservation Biology, and Biological Conservation to be applied journals; both applied and basic journals varied greatly in the prevalence of hypotheses

Counter to our hypothesis, applied and basic articles did not show a statistically significant difference in the prevalence of either mechanistic (GLMM: $\hat{\beta }$ = 0.054 [95% CI: −0.620, 0.728], *z* = 0.16, *p* = 0.875) or multiple alternative hypotheses (GLMM: $\hat{\beta }$ = 0.517 [95% CI: −0.582, 1.80], *z* = 0.88, *p* = 0.375). Although both basic and applied ecology and evolution articles containing hypotheses were similarly rare overall, there was a tendency for applied ecology articles to show increasing prevalence of mechanistic hypothesis use over time, whereas basic ecology articles have remained relatively unchanged (Table S1, Figure S1). However, there was substantial variation across both basic and applied journals in the prevalence of hypotheses (Figure 3).

### 3.2 Do hypotheses “pay?”

**FIGURE 4:**
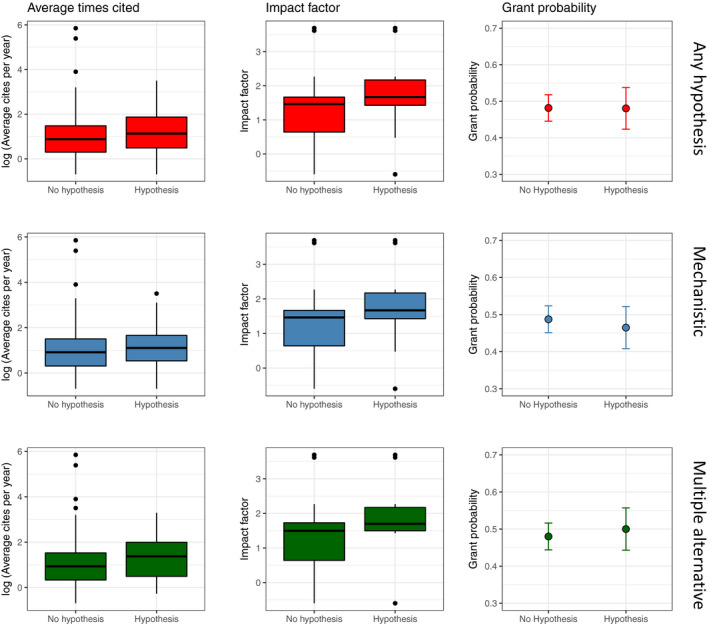
Results of our detailed literature search showing the relationship between having a hypothesis (or not) and three commonly sought after scientific rewards (Average times a paper is cited/year, Journal impact factor, and the likelihood of having a major national competitive grant). We found no statistically significant relationships between having a hypothesis and citation rates or grants, but articles with hypotheses tended to be published in higher impact journals

We found little evidence that presence of hypotheses increased paper citation rates. Papers with mechanistic (LMM: $\hat{\beta }$ = −0.109 [95% CI: −0.329, 0.115], *t* = 0.042, *p* = 0.97, Figure 4a, middle panel) or multiple alternative hypotheses (LMM: $\hat{\beta }$ = −0.008 [95% CI: −0.369, 0.391], *t* = 0.042, *p* = 0.96, Figure 4a, bottom panel) did not have higher average annual citation rates, nor did papers with at least one hypothesis type (LMM: $\hat{\beta }$ = −0.024 [95% CI: −0.239, 0.194], *t* = 0.218, *p* = 0.83, Figure 4a, top panel).

On the other hand, journal articles containing mechanistic hypotheses tended to be published in higher impact journals (GLM: $\hat{\beta }$ = 0.290 [95% CI: 0.083, 0.497], *t* = 2.74, *p* = 0.006) but only slightly so (Figure 4b, middle panel). Including multiple alternative hypotheses in papers did not have a statistically significant effect (GLM: = 0.339 [95% CI: −0.029, 0.707], *t* = 1.80, *p* = 0.072, Figure 4b, bottom panel).

Finally, we found no association between obtaining a competitive national or international grant and the presence of a hypothesis (logistic regression: mechanistic: $\hat{\beta }$ = −0.090 [95% CI: −0.637, 0.453], *z *= −0.36, *p* =0 .745; multiple alternative: $\hat{\beta }$ = 0.080 [95% CI: −0.891, 1.052], *z* = 0.49, *p* = 0.870; any hypothesis: $\hat{\beta }$ = −0.005 [95% CI: −0.536, 0.525], *z* = −0.02, *p* = 0.986, Figure 4c).

## 4 DISCUSSION

Overall, the prevalence of hypothesis use in the ecological and evolutionary literature is strikingly low and has been so for the past 25 years despite repeated calls to reverse this pattern (Elliott & Brook, 2007; Peters, 1991; Rosen, 2016; Sells et al., 2018). Why is this the case?

Clearly, hypotheses are not always necessary and a portion of the sampled articles may represent situations where hypotheses are truly not useful (see Box 3: “When Are Hypotheses Not Useful?”). Some authors (Wolff & Krebs, 2008) overlook knowledge gathering and descriptive research as a crucial first step for making observations about natural phenomena—from which hypotheses can be formulated. This descriptive work is an important part of ecological science (Tewksbury et al., 2014), but may not benefit from strict use of hypotheses. Similarly, some efforts are simply designed to be predictive, such as auto‐recognition of species via machine learning (Briggs et al., 2012) or for prioritizing conservation efforts (Wilson et al., 2006), where the primary concern is correct identification and prediction rather than the biological or computational reasons for correct predictions (Box 3). However, it would be surprising if 75% of ecology since 1990 has been purely descriptive work from little‐known systems or purely predictive in nature. Indeed, the majority of the articles we observed did not fall into these categories.

BOX 3 **When are hypotheses not useful?**

Of course, there are a number of instances where hypotheses might not be useful or needed. It is important to recognize these instances to prevent the pendulum from swinging in a direction where without hypotheses, research ceases to be considered science (Wolff & Krebs, 2008). Below are several important types of ecological research where formulating hypotheses may not always be beneficial.

**When the goal is prediction rather than understanding.** Examples of this exception include species distribution models (Elith et al., 2008) where the question is not why species are distributed as they are, but simply where species are predicted to be. Such results can be useful in conservation planning (Guisan et al., 2013; see below). Another example lies in auto‐recognition of species (Briggs et al., 2012) where the primary concern is getting identification right rather than the biological or computational reasons for correct predictions. In such instances, complex algorithms can be very effective at uncovering patterns (e.g., deep learning). A caveat and critical component of such efforts is to ensure that such models are tested on independent data. Further, if model predictions are made beyond the spatial or temporal bounds of training or test data, extreme caution should be applied (see Figure 4).

**When the goal is description rather than understanding.** In many applications, the objective is to simply quantify a pattern in nature; for example, where on Earth is forest loss most rapid (Hansen et al., 2013)? Further, sometimes so little is known about a system or species that formulating hypotheses is impossible and more description is necessary. In rare instances, an ecological system may be so poorly known and different to other systems that generating testable hypotheses would be extremely challenging. Darwin's observations while traveling on the Beagle are some of the best examples of such “hypothesis generating” science; these initial observations resulted in the formulation of one of the most extensively tested hypotheses in biology. However, such novelty should be uncommon in ecological and evolutionary research where theoretical and empirical precedent abounds (Sells et al., 2018). In the field of biogeography, there is the commonly held view that researchers should first observe and analyze patterns, and only then might explanations emerge (“pattern before process”); however, it has frequently been demonstrated that mechanistic hypotheses are useful even in disciplines where manipulative experiments are impossible (Crisp et al., 2011).

**When the objective is a practical planning outcome such as reserve design.** In many conservation planning efforts, the goal is not to uncover mechanisms, but rather simply to predict efficient methods or contexts for conserving species (Myers et al., 2000; Wilson et al., 2006). Perhaps this is the reason for such low prevalence of hypotheses in conservation journals (e.g., Conservation Biology).

Alternatively, researchers may not include hypotheses because they see little individual‐level incentive for their inclusion. Our results suggest that currently there are relatively few measurable benefits to individuals. Articles with mechanistic hypotheses do tend to be published in higher impact factor journals, which, for better or worse, is one of the key predictors in obtaining an academic job (van Dijk et al., 2014). However, few of the other typical academic metrics (i.e., citations or grant funding) appear to reward this behavior. Although hypotheses might be “useful” for overall progress in science (Platt, 1964), for their use to be propagated in the population of scientists, one would also expect them to provide benefits to the individuals conducting the science. Interestingly, the few existing papers on hypotheses (Loehle, 1987; Romesburg, 1981; Sells et al., 2018) tended to explain the advantages in terms of benefits to the group by offering arguments such as “because hypotheses help the field move forward more rapidly”.

Here we address some common justifications for hypotheses being unnecessary and show how one's first instinct to avoid hypotheses may be mistaken. We also present four reasons that use of hypotheses may be of individual self‐interest.

## 5 RESPONSES TO COMMON JUSTIFICATIONS FOR THE ABSENCE OF HYPOTHESES

During our collective mentoring at graduate and undergraduate levels, as well as examination of the literature, we have heard a number of common justifications for why hypotheses are not included. We must admit that many of us have, on occasion, rationalized absence of hypotheses in our own work using the same logic! We understand that clearly formulating and testing hypotheses can often be challenging, but propose that the justifications for avoiding hypotheses should be carefully considered.

1. “***But I do have hypotheses***”. Simply using the word “hypothesis” does not a hypothesis make. A common pattern in the literature we reviewed was for researchers to state their guess about the results they expect and call this the “hypothesis” (e.g., “I hypothesize trees at higher elevation will grow slowly”). But these are usually predictions derived from an implicit theoretical model (Symes et al., 2015) or are simply descriptive statements with the word “hypothesis” in front of them (see Box 1). A research hypothesis must contain explanations for an observed phenomenon (Loehle, 1987; Wolff & Krebs, 2008). Such explanations are derived from existing or new theory (Symes et al., 2015). Making the link between the expected mechanism (hypothesis) and logical outcome if that mechanism were true (the prediction), is a key element of strong inference. Similarly, using “statistical hypotheses” and “null hypothesis testing” is not the same as developing mechanistic research hypotheses (Romesburg, 1981; Sells et al., 2018).
2. “***Not enough is known about my system to formulate hypotheses***”. This is perhaps the most common defense against needing hypotheses (Golub, 2010). The argument goes that due to lack of previous research no mature theory has developed, so formal tests are impossible. Such arguments may have basis in some truly novel contexts (e.g., exploratory research on genomes) (Golub, 2010). But on close inspection, similar work has often been conducted in other geographic regions, systems, or with different taxa. If the response by a researcher is “but we really need to know if X pattern also applies in this region” (e.g., does succession influence bird diversity in forests of Western North America the same way as it does in Eastern forests), this is fine and it is certainly useful to accumulate descriptive studies globally for future synthetic work. However, continued efforts at description alone constitute missed opportunities for understanding the mechanisms behind a pattern (e.g., why does bird diversity decline when the forest canopy closes?). Often with a little planning, both the initial descriptive local interest question (e.g., “is it?”) and the broader interest question (i.e., “why?”) can both be tackled with minimal additional effort.
3. “***What about Darwin? Many important discoveries have been made without hypotheses***.” Several authors (and many students) have argued that many important and reliable patterns in nature have emerged outside of the hypothetico‐deductive (H‐D) method (Brush, 1974). For instance, Darwin's discovery of natural selection as a key force for evolution has been put forward as an example of how reliable ideas can emerge without the H‐D method (May, 1981; Milner, 2018). Examination of Darwin's notebooks has suggested that he did not propose explicit hypotheses and test them (Brush, 1974). However, Darwin himself wrote “all observation must be for or against some view if it is to be of any service!” (Ayala, 2009). In fact, Darwin actually put forward and empirically tested hypotheses in multiple fields, including geology, plant morphology and physiology, psychology, and evolution (Ayala, 2009). This debate suggests that, like Darwin, we should continue to value systematic observation and descriptive science (Tewksbury et al., 2014), but whenever possible, it should be with a view toward developing theory and testing hypotheses

The statement that “many important discoveries have been made without hypotheses” stems from a common misconception that somehow hypotheses spring fully formed into the mind, and that speculation, chance and induction play no role in the H‐D method. As noted by Loehle (1987; p. 402) “The H‐D method and strong inference, however, are valid no matter how theories are obtained. Dreams, crystal balls, or scribbled notebooks are all allowed. In fact, induction may be used to create empirical relations which then become candidates for hypothesis testing even though induction cannot be used to prove anything”. So, although induction has frequently been used to develop theory, it is an unreliable means to test theory (Popper, 1959). As is well‐known, Darwin's theory of natural selection was heavily debated in scientific circles at the time, and it is only through countless hypothesis tests that it remains the best explanation for evolution even today (Mayr,  2002).

- “***Ecology is too complex for hypotheses***”. In one of the most forcefully presented arguments for the H‐D method, Karl Popper (1959) argued that science should be done through a process of falsification; that is, multiple hypotheses should be constructed and the researcher's role is to successively eliminate these one at a time via experimentation until a single plausible hypothesis remains. This approach has caused some consternation among ecologists because the idea of single causes to phenomena doesn't match most of our experiences (Quinn & Dunham, 1983); rather, multiple interacting processes often overlap to drive observed patterns. For example, Robert Paine found that the distribution of a common seaweed was best explained by competition, physical disturbance, *and* dispersal ability (Paine, 1966).

It would be interesting if Popperian logic has inoculated ecology and evolution against the frequent application of hypotheses in research. Perhaps because the bar of falsification and testable mutually exclusive hypotheses is so high, many have opted to ignore the need for hypotheses altogether. If this is the case, our response is that in ecology and evolution we must not let Popperian perfection be the enemy of strong inference. With sufficient knowledge of a system, formal a priori hypotheses can be formulated that directly address the possibility of nonlinear relationships and interactions among variables. An example from conservation biology is the well‐explored hypothesis that the effects of habitat fragmentation should be greatest when habitat amount is low due to dispersal limitation (i.e., there should be a statistical interaction between fragmentation and habitat loss (Andrén, 1994)).

**FIGURE 5:**
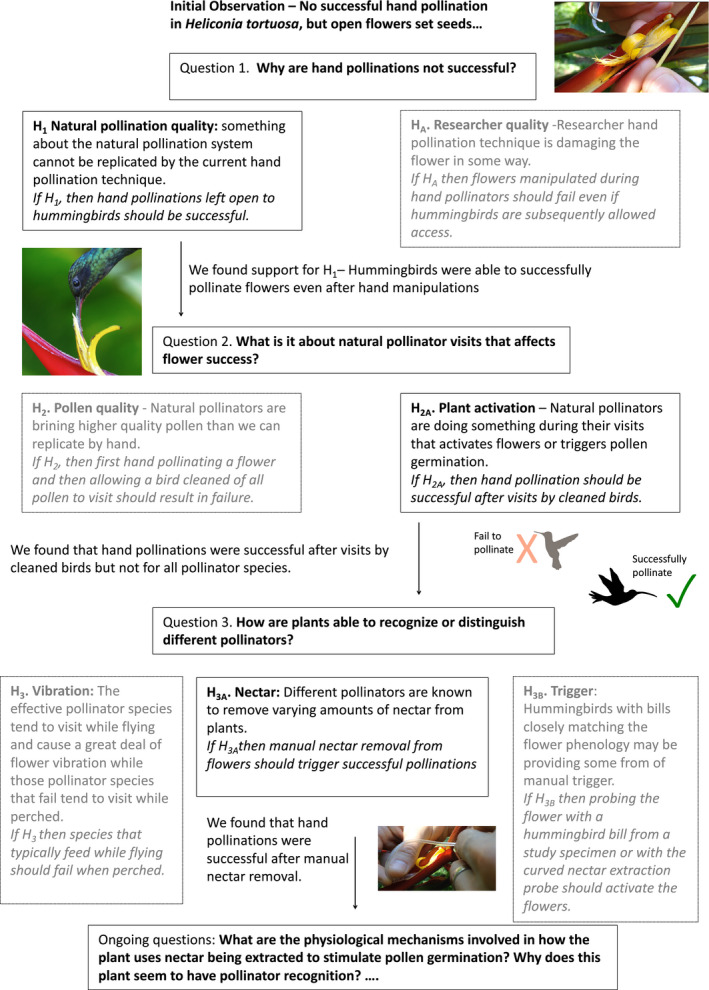
Hypothesis generation is possible at all levels of organization, and does not need to get to the bottom of a causal hierarchy to be useful. As illustrated in this case study (after Betts et al., 2015), using published work by the authors, support for a hypothesis at one level often generates a subsequent question and hypotheses at the next. After each new finding we had to return to the white board and draw out new alternative hypotheses as we progressed further down the hierarchy. Supported hypotheses are shown in black and the alternative hypotheses that were eliminated are in grey. A single study is not expected to tackle an entire mechanistic hierarchy. In fact, we still have yet to uncover the physiological mechanisms involved in this phenomenon

- “***But I am not a physiologist***.” A common misconception has to do with the hierarchical aspect of mechanisms (Figure 5). Many think that they are not testing the mechanism for a pattern because they have not managed to get to the bottom of a causal hierarchy (which reflects a sort of physics envy that commonly occurs in ecology and evolution (Egler, 1986)). However, hierarchy theory (O'Neill et al., 1989), states that the cause of a given phenomenon usually occurs at the level of organization just below the observed phenomenon. So, for example, species distributions might be best understood by examining hypotheses about the spatial composition and configuration of landscapes (Fahrig, 2003), explanations for population regulation might be best explored through observing the reproductive success and survival of individual organisms (Lack, 1954), and to understand individual variation in fecundity, one might test hypotheses relating to individual behavior or physiology. Hypothesis generation is possible at all levels of organization (Figure 5). Support for a hypothesis at one level often generates a subsequent question and hypotheses at the next (e.g., Observation: variation in animal densities can best be explained by forest patch size; Question: why are densities lower in small patches? H_1_: small patches have more edge, and predation rates are higher at the edge). However, in a single research project it is not necessary to develop hypotheses that address mechanisms at all scales.
- “***But my model predicts patterns well***”. An increasingly common justification for not presenting and testing research hypotheses seems to be the notion that if large datasets and complex modeling methods can predict outcomes effectively, what is the need for hypothesizing a mechanism (Glass & Hall, 2008; Golub, 2010)? Indeed, some have argued that prediction is a gold standard in ecology and evolution (Houlahan et al., 2017). However, underlying such arguments is the critical assumption that the relationship between predictors (i.e., independent variables, 'x's) and responses ('y's) exhibit *stationarity* in time and space. Although this appears to be the case in cosmology (e.g., relativity is thought to apply wherever you are in the universe (Einstein, 1920)), the assumption of stationarity has repeatedly been shown to be violated in ecological and evolutionary studies (Betts et al., 2006; Osborne et al., 2007; Thompson, 2005). Hence the well‐known maxim “correlation does not equal causation;” correlates of a phenomenon often shift, even if the underlying cause remains the same.

The advantage of understanding mechanism is that the relationship between cause and effect is less likely to shift in space and time than between the correlates of a phenomenon (Sells et al., 2018) (Figure 1). For instance, climate‐envelope models are still commonly used to predict future species distributions (Beale et al., 2008) despite the fact that links between correlates often fail (Gutiérrez et al., 2014) and climate per se may not be the direct driver of distributions. In an example from our own group, predictions that fit observed data well in the region where the model was built completely failed when predicted to a new region only 250 km away (Betts et al., 2006). Although it is true that mechanisms can also exhibit nonstationarity, at least in these instances logic can inform decisions about whether or not causal factors are likely to hold in a new place or time.

## 6 WHY SHOULD YOU HAVE HYPOTHESES? (A SELF‐INTERESTED PERSPECTIVE)

We have already described two arguments for hypothesis use, both of which should have positive influences on reproducibility and therefore progress in science: (1) multiple alternative hypotheses developed a priori prevent attachment to a single idea, and (2) hypotheses encourage exploration of mechanisms, which should increase the transferability of findings to new systems. Both these arguments have been made frequently in the eco‐evolutionary literature for decades (Elliott & Brook, 2007; Loehle, 1987; Rosen, 2016; Sells et al., 2018), but our results show that such arguments have been lost on the majority of researchers. One hypothesis recently proposed to explain why “poor methods persist [in science] despite perennial calls for improvements” is that such arguments have largely failed because they do not appeal to researcher self‐interest (Smaldino & McElreath, 2016). In periods of intense competition for grants and top‐tier publications, perhaps arguments that rely on altruism fall short. However, happily, there are at least four self‐interested reasons that students of ecological and evolutionary science should adopt the hypothetico‐deductive method.

1. **Clarity and Precision in Research**

First, and most apparent during our review of the literature, hypotheses force clarity and precision in thinking. We often found it difficult to determine the core purpose of papers that lacked clear hypotheses. One of the key goals of scientific writing is to communicate ideas efficiently (Schimel, 2011). Increased clarity through use of hypotheses could potentially even explain the pattern for manuscripts using hypotheses getting published in higher impact journals. Editors are increasingly pressed for time and forced to reject the majority of papers submitted to higher impact outlets prior to detailed review (AAAS, 2018). “Unclear message” and “lack of clear hypotheses” are top reasons a paper ends up in the editor's reject pile (Eassom, 2018; Elsevier, 2015). If editors have to struggle as often as we did to determine the purpose of a paper, this does not bode well for future publication. Clearly, communication through succinctly stated hypotheses is likely to enhance publication success.

Hypotheses also provide crucial direction during study design. Nothing is more frustrating than realizing that your hard‐earned data cannot actually address the key study objectives or rule out alternative explanations. Developing clear hypotheses and, in particular, multiple alternative hypotheses ensures that you actually design your study in a way that *can* answer the key questions of interest.

- **Personal Fulfillment**

Second, science is more likely to be fulfilling and fun when the direction of research is clear, but perhaps more importantly, when questions are addressed with more than one plausible answer. Results are often disappointing or unfulfilling when the study starts out with a single biological hypothesis in mind (Symes et al., 2015)—particularly if there is no support for this hypothesis. If multiple alternative hypotheses are well crafted, something interesting and rewarding will result regardless of the outcome. This results in a situation where researchers are much more likely to enjoy the *process* of science because the stress of wanting a particular end is removed. Subsequently, as Chamberlin (1890) proposed, “the dangers of parental affection for a favorite theory can be circumvented” which should reduce the risk of creeping bias. In our experience reviewing competitive grant proposals at the U.S. National Science Foundation, it is consistently the case that proposals testing several compelling hypotheses were more likely to be well received—presumably because reviewers are risk‐averse and understand that ultimately finding support for *any* of the outcomes will pay‐off. Why bet on just one horse when you can bet on them all?

- **Intrinsic Value to Mechanism**

Mechanism seems to have intrinsic value for humans—regardless of the practical application. Humans tend to be interested in acquiring understanding rather than just accumulating facts. As a species, we crave answers to the question “why.” Indeed, it is partly this desire for mechanism that is driving a recent perceived “crisis” in machine learning, with the entire field being referred to as “alchemy” (Hutson, 2018); algorithms continue to increase in performance, but the mechanisms for such improvements are often a mystery—even to the researchers themselves. “Because our model predicts well” is the unsatisfying scientific equivalent to a parent answering a child's “why?” with “because that's just the way it is.” This problem is beginning to spawn a new field in artificial intelligence “AI neuroscience” which attempts to get into the “black‐box” of machine‐learning algorithms to understand how and why they are predictive (Voosen, 2017).

Even in some of our most applied research, we find that managers and policymakers when confronted with a result (e.g., thinning trees to 70% of initial densities reduced bird diversity) want to know *why* (e.g., thinning eliminated nesting substrate for 4 species); If the answer to this question is not available, policy is much less likely to change (Sells et al., 2018). So, formulating mechanistic hypotheses will not only be more personally satisfying, but we expect it may also be more likely to result in real‐world changes.

- **You Are More Likely To be Right**

In a highly competitive era, it seems that in the quest for high publication rates and funding, researchers lose sight of the original aim of science: To discover a truth about nature that is transferable to other systems. In a recent poll conducted by *Nature,* more than 70% of researchers have tried and failed to reproduce another scientist's experiments (Baker, 2016). Ultimately, each researcher has a choice; put forward multiple explanations for a phenomenon on their own or risk “attachment” to a single hypothesis and run the risk of bias entering their work, rendering it irreproducible, and subsequently being found wrong by a future researcher. Imagine if Lamarck had not championed a single hypothesis for the mechanisms of evolution? Although Lamarck potentially had a vital impact as an early proponent of the idea that biological evolution occurred and proceeded in accordance with natural laws (Stafleu, 1971), unfortunately in the modern era he is largely remembered for his pet hypothesis. It may be a stretch to argue that he would have necessarily come up with natural selection, but if he *had* considered natural selection, the idea would have emerged 50 years earlier, substantially accelerating scientific progress and limiting his infamy as an early evolutionary biologist. An interesting contemporary example is provided by Prof. Amy Cuddy's research focused on “power posing” as a means to succeed. The work featured in one of the most viewed TED talks of all time but rather famously turned out to be irreproducible (Ranehill et al., 2015). When asked in a TED interview what she would do differently now, Prof. Cuddy noted that she would include a greater diversity of theory and multiple potential lines of evidence to “shed light on the psychological mechanisms” (Biello, 2017).

## 7 CONCLUSION

We acknowledge that formulating effective hypotheses can feel like a daunting hurdle for ecologists. However, we suggest that initial justifications for absence of hypotheses may often be unfounded. We argue that there are both selfish and altruistic reasons to include multiple alternative mechanistic hypotheses in your research: (1) testing multiple alternative hypotheses simultaneously makes for rapid and powerful progress which is to the benefit of all (Platt, 1964), (2) you lessen the chance that confirmation bias will result in you publishing an incorrect but provocative idea, (3) hypotheses provide clarity in design and writing, (4) research using hypotheses is more likely to be published in a high‐impact journal, and (5) you are able to provide satisfying answers to “why?” phenomena occur. However, few current academic metrics appear to reward use of hypotheses. Therefore, we propose that in order to promote hypothesis use we may need to provide additional incentives (Edwards & Roy, 2016; Smaldino & McElreath, 2016). We suggest editors reward research conducted using principles of sound scientific method and be skeptical of research that smacks of data dredging, post hoc hypothesis development, and single hypotheses. If no hypotheses are stated in a paper and/or the paper is purely descriptive, editors should ask whether the novelty of the system and question warrant this, or if the field would have been better served by a study with mechanistic hypotheses. Eleven of the top 20 ecology journals already indicate a desire for hypotheses in their instructions for authors—with some going as far as indicating “priority will be given” for manuscripts testing clearly stated hypotheses. Although hypotheses are not necessary in all instances, we expect that their continued and increased use will help our disciplines move toward greater understanding, higher reproducibility, better prediction, and more effective management and conservation of nature. We recommend authors, editors, and readers encourage their use (Box 2).

## CONFLICT OF INTEREST

The authors have no conflicts of interests to declare.

## AUTHOR CONTRIBUTIONS

**Matthew G. Betts:** Conceptualization (lead); data curation (lead); formal analysis (lead); funding acquisition (lead); investigation (lead); methodology (equal); project administration (lead); resources (lead); supervision (lead); visualization (lead); writing‐original draft (lead); writing‐review & editing (lead). **Adam S. Hadley:** Conceptualization (lead); data curation (lead); funding acquisition (equal); investigation (equal); methodology (lead); project administration (equal); resources (supporting); software (supporting); supervision (lead); validation (lead); visualization (lead); writing‐original draft (equal); writing‐review & editing (equal). **David W. Frey:** Conceptualization (supporting); data curation (supporting); formal analysis (supporting); funding acquisition (supporting); writing‐review & editing (supporting). **Sarah J. K. Frey:** Conceptualization (supporting); Investigation (equal); writing‐review & editing (equal). **Dusty Gannon:** Conceptualization (supporting); Investigation (equal); writing‐review & editing (equal). **Scott H. Harris:** Conceptualization (supporting); Investigation (equal); methodology (equal); writing‐review & editing (equal). **Hankyu Kim:** Conceptualization (supporting); Investigation (equal); Methodology (equal); writing‐review & editing (equal). **Kara Leimberger:** Conceptualization (supporting); Investigation (equal); Methodology (equal); writing‐review & editing (equal). **Katie Moriarty:** Conceptualization (supporting); Investigation (equal); methodology (equal); writing‐review & editing (equal). **Joseph M. Northrup:** Investigation (equal); methodology (equal); writing‐review & editing (equal). **Ben Phalan:** Investigation (equal); Methodology (equal); writing‐review & editing (equal). **Josée S. Rousseau:** Investigation (equal); Methodology (equal); writing‐review & editing (equal). **Thomas D. Stokely:** Investigation (equal); methodology (equal); writing‐review & editing (equal). **Jonathon J. Valente:** Investigation (equal); methodology (equal); writing‐review & editing (equal). **Urs G. Kormann:** Methodology (supporting); resources (equal); writing‐review & editing (supporting). **Chris Wolf:** Formal analysis (supporting); writing‐review & editing (supporting). **Diego Zárrate‐Charry:** Investigation (equal); Methodology (equal); writing‐review & editing (equal).

## ETHICAL APPROVAL

The authors adhered to all standards for the ethical conduct of research.

## Supporting information

Supplementary Material

## Data Availability

Data for the analysis of hypothesis use in ecology and evolution publications is available at https://figshare.com/articles/dataset/Betts_et_al_2021_When_are_hypotheses_useful_in_ecology_and_evolution_Ecology_and_Evolution/14110289.
