# Supplementary material for: When are hypotheses useful in ecology and evolution?
Source: Ecol Evol. 2021 Mar 25;11(11):5762–76. doi: 10.1002/ece3.7365 (PMC8207363; doi:10.1002/ece3.7365)
Supplement: Supplementary file 1 — Supplementary Material [file ECE3-11-5762-s001.docx]

Betts et al.

Supporting Material

Table S1. Generalized Linear Mixed Model (GLMM) results testing whether 25-year trends in hypothesis usage differs between applied and basic research articles. The prevalence of articles with no hypotheses tended to decline for applied articles (indicated by the negative coefficient for ‘publication year’).

|  | $\hat{\beta}$ | SE | Z | *P* |
| --- | --- | --- | --- | --- |
| *Mechanistic hypothesis* |  |  |  |  |
| Intercept (‘applied’ reference) | -1.302 | 0.319 | -4.077 | 0.000 |
| Publication year | 0.467 | 0.288 | 1.620 | 0.105 |
| Basic | 0.193 | 0.362 | 0.534 | 0.593 |
| Year x basic | -0.458 | 0.337 | -1.360 | 0.174 |
| *Multiple hypotheses* |  |  |  |  |
| Intercept | -3.732 | 0.902 | -4.139 | 0.000 |
| Publication year | 1.235 | 0.778 | 1.588 | 0.112 |
| Basic | 1.229 | 0.945 | 1.301 | 0.193 |
| Year x basic | -1.352 | 0.827 | -1.635 | 0.102 |
| *No hypotheses* |  |  |  |  |
| Intercept | 1.078 | 0.319 | 3.378 | 0.001 |
| Publication year | -0.658 | 0.286 | -2.300 | 0.021 |
| Basic | 0.002 | 0.364 | 0.007 | 0.995 |
| Year x basic | 0.648 | 0.334 | 1.941 | 0.052 |

Fig. S1 Trends in hypothesis use in applied (red lines) versus basic (blue lines) in ecology and evolution journals from 1991-2015 from a sample of the ecological and evolutionary literature (A: descriptive hypotheses, B: mechanistic hypotheses, C: descriptive hypotheses [predictions], and D: no hypotheses present). Although the statistical interaction between ‘year’ and ‘applied versus basic’ was not statistically significant for any hypothesis type (at p<0.05) the incidence of articles containing no hypothesis at all (mechanistic, multiple alternative, or descriptive) shows a promising decline in the applied ecology literature (D).
